# Supplementary material for: Modeling glioblastoma heterogeneity as a dynamic network of cell states
Source: Mol Syst Biol. 2021 Sep 16;17(9):e10105. doi: 10.15252/msb.202010105 (PMC8444284; doi:10.15252/msb.202010105)
Supplement: Supplementary file 6 — Source Data for Figure 5 [file MSB-17-e10105-s004.zip › Figure5A_sourcedata/GSEA_3017/hallmarks_stateE.GseaPreranked.1621934677158/HALLMARK_GLYCOLYSIS.html]

Details for gene set HALLMARK\_GLYCOLYSIS[GSEA]

|  || Dataset | state63017 |
| Phenotype | NoPhenotypeAvailable |
| Upregulated in class | na\_neg |
| GeneSet | HALLMARK\_GLYCOLYSIS |
| Enrichment Score (ES) | -0.1935559 |
| Normalized Enrichment Score (NES) | -0.75597036 |
| Nominal p-value | 0.75857687 |
| FDR q-value | 0.7713186 |
| FWER p-Value | 1.0 |
Table: GSEA Results Summary

  

Fig 1: Enrichment plot: HALLMARK\_GLYCOLYSIS      
 Profile of the Running ES Score & Positions of GeneSet Members on the Rank Ordered List

  

| PROBE | GENE SYMBOL | GENE\_TITLE | RANK IN GENE LIST | RANK METRIC SCORE | RUNNING ES | CORE ENRICHMENT || 1 | CD44 |  |  | 14 | 0.806 | 0.0547 | No |
| 2 | GPC1 |  |  | 50 | 0.530 | 0.0626 | No |
| 3 | HSPA5 |  |  | 73 | 0.449 | 0.0777 | No |
| 4 | IRS2 |  |  | 85 | 0.426 | 0.1027 | No |
| 5 | VLDLR |  |  | 87 | 0.419 | 0.1379 | No |
| 6 | SDC3 |  |  | 200 | 0.295 | 0.0419 | No |
| 7 | IDH1 |  |  | 218 | 0.284 | 0.0481 | No |
| 8 | SDC2 |  |  | 226 | 0.280 | 0.0648 | No |
| 9 | GPC4 |  |  | 246 | 0.264 | 0.0670 | No |
| 10 | CHPF |  |  | 269 | 0.251 | 0.0649 | No |
| 11 | NASP |  |  | 410 | -0.285 | -0.0623 | No |
| 12 | EGFR |  |  | 427 | -0.291 | -0.0545 | No |
| 13 | HOMER1 |  |  | 463 | -0.304 | -0.0661 | No |
| 14 | CLN6 |  |  | 470 | -0.305 | -0.0462 | No |
| 15 | POLR3K |  |  | 474 | -0.307 | -0.0228 | No |
| 16 | KIF20A |  |  | 523 | -0.321 | -0.0471 | No |
| 17 | SAP30 |  |  | 558 | -0.339 | -0.0547 | No |
| 18 | PAXIP1 |  |  | 570 | -0.344 | -0.0368 | No |
| 19 | MIF |  |  | 580 | -0.349 | -0.0163 | No |
| 20 | PGAM1 |  |  | 670 | -0.410 | -0.0774 | No |
| 21 | STMN1 |  |  | 778 | -0.529 | -0.1477 | Yes |
| 22 | CENPA |  |  | 782 | -0.538 | -0.1043 | Yes |
| 23 | DEPDC1 |  |  | 815 | -0.596 | -0.0874 | Yes |
| 24 | AURKA |  |  | 824 | -0.623 | -0.0420 | Yes |
| 25 | HMMR |  |  | 863 | -0.740 | -0.0192 | Yes |
| 26 | CDK1 |  |  | 938 | -1.248 | 0.0087 | Yes |
Table: GSEA details [plain text format]

  

Fig 2: HALLMARK\_GLYCOLYSIS: Random ES distribution      
 Gene set null distribution of ES for **HALLMARK\_GLYCOLYSIS**
